# Why an extended evolutionary synthesis is necessary

**DOI:** 10.1098/rsfs.2017.0015

**Published:** 2017-08-18

**Authors:** Gerd B. Müller

**Affiliations:** 1 Department of Theoretical Biology, University of Vienna, Vienna, Austria; 2 Konrad Lorenz Institute for Evolution and Cognition Research, Klosterneuburg, Austria

**Keywords:** evolutionary biology, modern synthesis, extended synthesis, evolutionary developmental biology, niche construction, systems biology

## Abstract

Since the last major theoretical integration in evolutionary biology—the modern synthesis (MS) of the 1940s—the biosciences have made significant advances. The rise of molecular biology and evolutionary developmental biology, the recognition of ecological development, niche construction and multiple inheritance systems, the ‘-omics’ revolution and the science of systems biology, among other developments, have provided a wealth of new knowledge about the factors responsible for evolutionary change. Some of these results are in agreement with the standard theory and others reveal different properties of the evolutionary process. A renewed and extended theoretical synthesis, advocated by several authors in this issue, aims to unite pertinent concepts that emerge from the novel fields with elements of the standard theory. The resulting theoretical framework differs from the latter in its core logic and predictive capacities. Whereas the MS theory and its various amendments concentrate on genetic and adaptive variation in populations, the extended framework emphasizes the role of constructive processes, ecological interactions and systems dynamics in the evolution of organismal complexity as well as its social and cultural conditions. Single-level and unilinear causation is replaced by multilevel and reciprocal causation. Among other consequences, the extended framework overcomes many of the limitations of traditional gene-centric explanation and entails a revised understanding of the role of natural selection in the evolutionary process. All these features stimulate research into new areas of evolutionary biology.

## 1. Introduction

A century ago, it was noted in the domain of physics that ‘concepts that have proven useful in ordering things easily achieve such an authority over us that we forget their earthly origins and accept them as unalterable givens. Thus, they come to be stamped as “necessities of thought”, “*a priori* givens”, etc. The path of scientific advance is often made impassable for a long time through such errors.’ [1]. Evolutionary biology finds itself in a similar situation today. A well-established paradigm that has its roots in a major theoretical integration that took place approximately eight decades ago, traditionally labelled the modern synthesis (MS) or Synthetic Theory, still dominates evolutionary thought today. In the meantime, the biological sciences have progressed extensively. The material basis of inheritance has been unravelled and entire new fields of research have arisen, such as molecular genetics, evolutionary developmental biology and systems biology. In addition, new evolutionarily relevant factors have been described, including non-genetic inheritance, developmental bias, niche construction, genomic evolution and others. Clearly, our understanding of evolution has significantly expanded, and it would be surprising if these empirical and conceptual advances had no theoretical consequences, so that in the midst of a substantial growth of knowledge, the central theory uniting the different fields of biology remained unaltered.

In fact, our theoretical understanding of biological evolution has not remained unaltered. Slight modifications and adjustments to the received theory are recognized even in the most traditional quarters. But in the past decade, without much notice by general audiences, a more wide-ranging debate has arisen from different areas of biology as well as from history and philosophy of science, about whether and in which ways evolutionary theory is affected, challenged or changed by the advances in biology and other fields. As usual in such cases, more conservative perspectives and more progressive ones are in conflict with each other, with differences ranging from minor to intense. A rising number of publications argue for a major revision or even a replacement of the standard theory of evolution [2–14], indicating that this cannot be dismissed as a minority view but rather is a widespread feeling among scientists and philosophers alike. In the present essay, I will concentrate on the arguments and debates triggered by one particular alternative to the standard theory that has become known under the term extended evolutionary synthesis (EES). This proposal for an integration of revised and additional components of evolutionary theory into a coherent explanatory framework, as recently elaborated by Laland *et al*. [15], has caught on as one of the crystallizing points in the ongoing debate. No claim is made that this approach represents the only way of addressing theory revision in biology.

The theory of evolution is the fundamental conceptual framework of biology all scientific explanations of living phenomena must be consistent with. As it does not describe a universal law regarding a single natural phenomenon, such as gravity, but rather the principles of organismal change over time, based on the highly complex inputs and interactions of a multiplicity of different factors, evolutionary theory cannot be expected to remain static but is subject to change in the light of new empirical evidence. This is a normal process of scientific advancement and not a heretical undertaking as it is sometimes perceived to be. Explanations of organismal diversity have changed significantly during pre- and post-Darwinian periods, and it should not come as a surprise that fresh stimuli arise from the new methodologies and the expanded scope of modern biological research. Indeed, a growing number of challenges to the classical model of evolution have emerged over the past few years, such as from evolutionary developmental biology [16], epigenetics [17], physiology [18], genomics [19], ecology [20], plasticity research [21], population genetics [22], regulatory evolution [23], network approaches [14], novelty research [24], behavioural biology [12], microbiology [7] and systems biology [25], further supported by arguments from the cultural [26] and social sciences [27], as well as by philosophical treatments [28–31]. None of these contentions are unscientific, all rest firmly on evolutionary principles and all are backed by substantial empirical evidence.

Sometimes these challenges are met with dogmatic hostility, decrying any criticism of the traditional theoretical edifice as fatuous [32], but more often the defenders of the traditional conception argue that ‘all is well’ with current evolutionary theory, which they see as having ‘co-evolved’ together with the methodological and empirical advances that already receive their due in current evolutionary biology [33]. But the repeatedly emphasized fact that innovative evolutionary mechanisms have been mentioned in certain earlier or more recent writings does not mean that the formal structure of evolutionary theory has been adjusted to them. To the contrary, the discrepancies between the current usage of evolutionary concepts and the predictions derived from the classical model have grown. Hence, it will be useful to characterize some of the differences that exist between the MS theory and proposed alternatives.

## 2. A problem agenda

When attempting to define the issues at stake in the current debate, it is necessary to keep in sight what it is that should be explained by a theory of evolution. Evolutionary biology, as practised today, does not represent a single coherent approach but includes sets of different topics and research programmes. For instance, one may be interested in the patterns of phylogenetic relatedness and the processes of species formation. Here, the emphasis is on reconstructing relationships among organisms and unravelling the principles of the separation and diversification of higher taxonomical clades, as pursued by the field of phylogenetics. Another approach examines genetic and phenotypic variation in populations in order to derive rules of variational change over time, a perspective most elaborately pursued by population genetics and quantitative genetics. Still another approach is the study of the origin of complex organismal features, such as morphological, physiological or behavioural traits, in order to explain the evolution of the processes that generate these features and how—in turn—these processes influence the course of evolution, as investigated by evolutionary developmental biology (evo-devo), systems biology, or the behavioural sciences. Finally, one may study evolution with a view to the origins of mind, language, society and culture, as well as their feedback on biological evolution, as conducted by the fields of cognitive biology, linguistics, anthropology and certain domains of the social sciences.

The vastly different explananda and the progress made in each of these fields must be kept in mind when we examine the tenets of the present theory of evolution. While documenting numerous empirical and theoretical advances, at the level of core assumption most current textbooks on evolution, whether explicitly or implicitly, still offer a theoretical framework that is largely based on the MS of the 1930s and 1940s. Even though it never constituted an encompassing formal synthesis [34], this movement had brought together the basic neo-Darwinian principles of variation, inheritance, differential reproduction and natural selection with Mendelian, experimental and population genetics, as well as with concepts and data addressing the patterns of evolution stemming from the fields of palaeontology, botany and systematics. The formalized core of the MS theory was—and still is—population genetics [35], a mathematical account of gene frequency dynamics in populations of organisms. The empirical basis and key concern of the population genetic approach is the measurement of trait variability in populations, and its intended explananda are adaptive variation, speciation and calculations of fitness. The flurry of fitness landscapes based on ever more nuanced algorithms is indicative of this received approach.

Even though claims have been made that classical evolutionary biology has continuously incorporated aspects from new conceptual domains [33,36], the majority of tenets and explanations that appear in characterizations of the current theory are still derived from the MS account and its population genetic principles [37]. In a condensed form, these tenets are as follows: (i) all evolutionary explanation requires the study of populations of organisms; (ii) populations contain genetic variation that arises randomly from mutation and recombination; (iii) populations evolve by changes in gene frequency brought about by natural selection, gene flow and drift; (iv) genetic variants generate slight phenotypic effects and the resulting phenotypic variation is gradual and continuous; (v) genetic inheritance alone accounts for the transmission of selectable variation; (vi) new species arise by a prevention of gene flow between populations that evolve differently; (vii) the phenotypic differences that distinguish higher taxa result from the incremental accumulation of genetic variation; (viii) natural selection represents the only directional factor in evolution. For a more extensive description of tenets see Futuyma [37].

As can be noted from the listed principles, current evolutionary theory is predominantly oriented towards a genetic explanation of variation, and, except for some minor semantic modifications, this has not changed over the past seven or eight decades. Whatever lip service is paid to taking into account other factors than those traditionally accepted, we find that the theory, as presented in extant writings, concentrates on a limited set of evolutionary explananda, excluding the majority of those mentioned among the explanatory goals above. The theory performs well with regard to the issues it concentrates on, providing testable and abundantly confirmed predictions on the dynamics of genetic variation in evolving populations, on the gradual variation and adaptation of phenotypic traits, and on certain genetic features of speciation. If the explanation would stop here, no controversy would exist. But it has become habitual in evolutionary biology to take population genetics as the privileged type of explanation of *all* evolutionary phenomena, thereby negating the fact that, on the one hand, not all of its predictions can be confirmed under all circumstances, and, on the other hand, a wealth of evolutionary phenomena remains excluded. For instance, the theory largely avoids the question of how the complex organizations of organismal structure, physiology, development or behaviour—whose variation it describes—actually arise in evolution, and it also provides no adequate means for including factors that are not part of the population genetic framework, such as developmental, systems theoretical, ecological or cultural influences.

Criticisms of the shortcomings of the MS framework have a long history. One of them concerns the profoundly gradualist conception the MS has inherited from the Darwinian account of evolution. Darwin saw slight, incremental and accumulating variation as *the* essential prerequisite without which ‘my theory would absolutely break down.’ [38] a position already characterized by Huxley in 1901 [39] as an ‘unnecessary difficulty.’ Subsequently, the perceived necessity of a slow and continuous flux of variation seemed to have been supported by innumerable studies that demonstrate corresponding behaviours of character variation in natural populations or under artificial selection regimes. The notion of slight successive variation was further reinforced by the molecular conception of genetic variation. When mutation of individual genes or even smaller entities of DNA is taken as the predominant source of variation, it seemed inevitable that phenotypic modifications should be small, because larger changes were deemed to be disruptive and unlikely to produce adaptive outcomes. The supposed randomness of genetic variation further contributed to this view. Today, all of these cherished opinions have to be revised, not least in the light of genomics, which evokes a distinctly non-gradualist picture [40]. In addition, it is necessary to realize that all models of gradual variation are based on empirical measurements of precisely this kind of change and to the exclusion of other forms of variation. If cases of gradual variation are chosen and quantified, and theoretical models are derived from them, it should not be unexpected that it is gradual variation that will be explained.

Connected with the gradualist requirement of the MS theory is the deeply entrenched notion of adaptation. Again, we are confronted with a feature of the classical theory that has been criticized repeatedly in the past, both on empirical and theoretical grounds [30,41] but also on the basis of modern results of genetics [22]. Whereas different forms of adaptationism can be discerned, for instance in the British and the American research traditions [30], the notion most frequently encountered is still that of a collection of features that make up the organism, each one individually adapted to performing a function in the way best suited for the organism's survival, a picture that has been described as ‘bundles of discrete adaptations.’ This view was neither eliminated by Dobzhansky's alternative view, in which he interpreted populations as states of relative adaptedness [30], nor by the demonstration of the frequent occurrence of non-adaptive traits. Already in the late 1970s, Gould & Lewontin [41] described the adherence to pervasive adaptationism as an ‘old habit,’ but despite extensive learned discussions of the subject that habit has not receded.

Natural selection, the cornerstone of the MS theory so intimately linked to both gradualism and adaptationism, has itself been the subject of a fair share of critical debate. In this case, it is not so much the principle itself that is contested, but the uniqueness of the causal agency that has been ascribed to it. Are all features of biological organisms necessarily the result of natural selection, and is it the only factor in the evolutionary process that provides directionality to organismal change? Numerous authors have challenged the pervasiveness of natural selection as a unique ‘force’ of evolution, whereas others have questioned whether the individual is the sole and appropriate ‘target’ of selection or whether other levels of selection at supra- and infra-individual levels also need to be included in selectionist scenarios [42–44]. Again we are confronted with a classical criticism that stood at the centre of multiple debates in the past [42], but the issue is as unresolved as ever.

Finally, it is apparent that nearly all of the relevant predictions that derive from the MS theory are based on genetic principles and gene determinist convictions. Although the long-held belief that genes are the unique determinants of biological form in development and evolution has been challenged by an extensive number of commentators [21,23,45–48], the genetic program idea underlying MS theory has remained unaltered. This is also true with regard to the mechanisms of transgenerational inheritance. The proposition of uniquely genetic inheritance has been falsified multiple times [3], but the gene-centric position remains constitutive of the MS. The contemporary version of this position, gene regulatory network evolution, really represents only an extension of the ‘gene-determines-phenotype’ view.

The limitations of the MS theory are not only highlighted by the criticisms directed against several of its traditional tenets but also by the failure to address some of the most important phenomena of organismal evolution. The question, for instance, of how complex phenotypic organizations arise in evolution is sidestepped by the population theoretical account, as is the reciprocal influence of these features of higher levels of organization on the evolutionary process. Indeed, the MS theory lacks a theory of organization that can account for the characteristic features of phenotypic evolution, such as novelty, modularity, homology, homoplasy or the origin of lineage-defining body plans. As will be shown below, evo-devo, niche construction, systems biology and other areas harbour the capacity to address at least certain aspects of these topics where the classical theory fails.

Even though only the most prominent issues were mentioned here, this brief overview indicates that the problem agenda associated with the MS theory is extensive. The fact, often mentioned by defenders of the orthodoxy, that these issues have been raised before, does not alleviate the problems. Rather, the current evolutionary paradigm is still dominated by the very same basic assumptions that marked the origin of the synthesis approach. Despite the fact that substantial challenges to these positions have arisen in the past decades from a host of different areas of biology, they have rarely resulted in alternative proposals. Gould's 2002 comprehensive treatment of the history of evolutionary debate [42], for instance, takes up most of the criticisms and suggests alternate concepts, but it does not actually offer an alternative overall *structure* of evolutionary theory as its title suggests. All the extensive discussions, led over decades, seem not to have altered the preponderant stance to hold on to the classical prerequisites of gradualism, adaptationism, selectionism and gene-centrism. The predictions that follow from the MS framework continue to be based on these prerequisites and ignore all predictions derived from alternative models. Hence, the claim of continuous incorporation of new conceptual components by the MS theory is misleading.

## 3. Conceptual innovation

Today, evolutionary biology exhibits a very different landscape. An abundance of new theoretical concepts has arisen since the time of the formulation of the population theoretical synthesis, some of which offer challenges to the received theory or have not been included into a common theoretical framework. Only a brief overview of the most relevant conceptual innovations is possible in the present context. For more elaborate treatments see Pigliucci & Müller [49] or Laland *et al*. [15].

### 3.1. Evolutionary developmental biology

**Figure 1.**
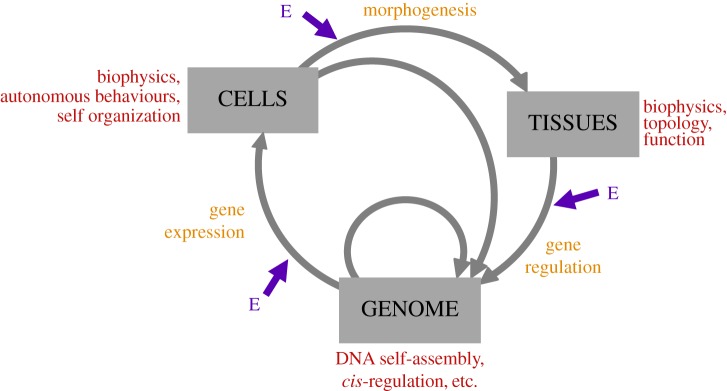
Feedback interactions among different levels of organization in developmental systems. Examples of autonomous properties of each level are marked in red (E, environmental influences).

A suite of new concepts emerges from evo-devo, a field of research that arose in the early 1980s from a discontent with the exclusion of developmental biology from evolutionary theory [50–53]. The subsequent rise of new molecular methodologies for a comparative analysis of gene regulation resulted in a huge increase of our understanding of how the processes of development evolve. In its theoretical domain, the evo-devo approach starts from the premise that the genotype–phenotype relation is not merely a statistical correlation, but that the rules of developmental processes govern phenotypic outcomes while relying on additional inputs not coming from the genome. It is abundantly clear that development is not a linear reading out of a code or program but a systemic process of feedback interactions between genetic and non-genetic templates, cells and tissues that mobilizes physical and autonomous properties at different scales and depends on local as well as global environments [54] (figure 1). Hence, development is a systems relation in which no component is informationally privileged. A number of evolutionary concepts result from the evo-devo study of these relations, three of which shall be mentioned here.

First, the kind of selectable phenotypic variation that can be produced by a developmental system of a given type is neither infinite nor random. Rather, selectable variation is both constrained [55] and facilitated [56] by development. Before natural selection can act, the developmental system harbours tendencies towards certain solutions, a property that has been called developmental bias [57,58]. Second, as is the case with most multilevel systems, developmental processes exhibit emergent properties. A wide array of such behaviours is known in cell and tissue organization [59]. Reaction–diffusion processes in embryos, for instance, organize cell arrangements in limb morphogenesis [60]. Third, developmental systems are characterized by bistabilities and threshold behaviours [61], such as in the well-known case of somite formation, in which there exists a mutually inhibiting relationship between FGF and RA receptors in which an unstable state separates two stable states [62], or in the case of threshold behaviours in vertebrate digit formation [63]. When natural selection affects such kinds of systems, the resulting phenotype variation does not need to be gradual and continuous. In fact, simulations of the dynamical behaviours of gene regulatory networks in evolution demonstrate that bistable changes are more likely to occur than gradual transitions [64].

In addition, the research approach of evo-devo permits addressing the processes responsible for the evolution of phenotypic organization, a topic thoroughly avoided by the synthesis theory. The issue cannot be reduced to the evolution of gene regulation, because, on the one hand, highly conserved developmental control genes, e.g. homeotic genes, can exhibit non-homologous expression domains in embryos of closely related phylogenetic lineages, and on the other hand, homologous structures can be specified by non-homologous genes, a characteristic of the genotype–phenotype relation described by developmental systems drift [65]. Evo-devo-based concepts of structural organization emphasize the integrative stability provided by shared developmental pathways [66] and the modularity of developmental processes [67]. Morphological templates that result from the mobilization of physical forces are seen to represent basic organizing themes in animals [68] and plants [69] that become integrated through a hierarchization of regulatory networks and fixated as patterns of phenotypic construction [70]. Increasingly elaborate gene regulatory systems serve to reproduce morphological templates, and the close mapping between genotype and morphological phenotype may not represent the cause but a consequence of evolution [71]. Hence, evo-devo mechanisms of phenotypic organization could not only be responsible for higher-level complexity but could also affect further organismal evolution [4], a claim that is supported by experiment [72] and modelling [73].

Overall, the evo-devo results indicate that phenotypic variation is neither necessarily gradual nor random. Irrespective of whether they are perturbed by selectional, mutational or experimental intervention, developmental systems exhibit emergent behaviours and generate nonlinear effects, i.e. the phenotypic outcome is only indirectly related to genetic variation and yet still follows predictable pathways. In other words, the variational range of a population is not defined merely by genetic variation but by the developmental system as a whole, providing sources of phenotypic bias and novelty [24,74]. It is now possible to determine the relative importance of natural selection and of genetic and developmental determinants of organic diversity [55].

### 3.2. Phenotypic plasticity

The population context of development is mostly provided by the study of developmental plasticity, a component of phenotypic plasticity. Developmental plasticity is the capacity of organisms to develop altered phenotypes in reaction to different environmental conditions. Among its evolutionary effects, the influence on a population's variational response to selection and the acceleration of the colonization of novel environments are well documented [21,75,76], as are the fitness consequences of parental effects that depend on the modifications of developmental processes [77]. Plasticity can also have a critical role in determining which genetic variants will generate selectable phenotypic differences under given environmental conditions, or as a result of stress [78], through either widening or narrowing the range of the phenotypic response capacity of a population, often termed the reaction norm [79].

According to the developmental plasticity concept, fixation of environmentally induced variants may happen through phenotypic and genetic accommodation [74,80]. Phenotypic accommodation refers to the adjustment of modified parts of an organism through developmental processes that typically do not require genetic mutation [81]. Phenotypic accommodation may be followed by genetic accommodation and thus result in more rapid adaptation to novel environments [79]. The effect of a novel environment on phenotypic plasticity may be coupled with simultaneous exposure of ‘hidden’ developmental variation and strong selection on this variation [82] and could provide a starting point for the evolutionary persistence of phenotypic innovations [24]. Plasticity has also been linked to the ubiquitous phenomenon of homoplasy [83] and to rapid divergence of phylogenetic lineages [21]. In this perspective, developmental plasticity acts as a source of adaptive innovation, and one of its critical mechanisms is environmental induction [21,82,84], the direct action of environmental parameters on developmental processes.

### 3.3. Genomics

The science most central to the MS, genetics, likewise has substantially changed since the time of the synthesis and especially over the past two decades. Now that whole genomes can be studied, we have learned that in the course of evolution significant portions of the genome have been duplicated, deleted or co-opted into new functions [40]. In addition, novel genomic segments and biochemical functions can be acquired from other cells and organisms, rather than exclusively by inheritance from their progenitors. Comparative genomics has greatly changed the concepts of both the evolution of primitive life forms and eukaryotes. Among prokaryotes, viruses, plasmids, etc., horizontal gene transfer is ubiquitous and even among eukaryotes much more frequent than hitherto assumed [85,86], with compelling documentations of horizontal transfer in protists, fungi and plants, as well as in animals, including mammals and other tetrapods [7]. Mobile elements, in particular, make genomic evolution exquisitely dynamic and non-gradual [40,87]. Furthermore, functional genome reorganization can occur in response to environmental stress [14,88–90]. Thus, the properties of genetic change are found to be quite different from the assumptions made by the founders of the MS, when continued random substitution of individual alleles was the reigning understanding.

### 3.4. Multilevel selection

Conceptual change is also underway with regard to the understanding of natural selection. Whereas the classical view posited the individual as the unit of selection, it is now held more often that natural selection can also act at levels above and below the individual. Hierarchical selection theory [42] and multilevel selection theory [69,91] span selective processes from genetic, cellular and tissue levels up to kin selection, group selection and possibly even species selection, making it necessary to distinguish individual fitness from group fitness. Even though the debate is still in flux, increasing attention is paid to the fact that natural selection may act at different levels simultaneously, possibly even in opposing directions, and that selection at one level can have effects that percolate up or down to other levels. Interest in multilevel selection theory has resurged in connection to work on the major transitions in evolution and definitions of biological causality [91].

### 3.5. Inclusive inheritance

The concepts of inheritance equally have undergone revision in recent decades. In addition to genetic inheritance, the only means of transgenerational transmission of information acknowledged by the MS, several forms of non-genetic inheritance are recognized today. These include epigenetic, behavioural, ecological and cultural forms of inheritance [3,9].

In the domain of epigenetic inheritance it is not merely the well-known patterns of post-translational modifications of histone proteins or methylation of cytosines that can be transmitted across generations [92]. Especially the transgenerational epigenetics of small RNAs is increasingly identified as a major factor of gene regulation in developing tissues, with a host of new molecules and mechanisms uncovered in recent times [93], including ways in which parental experience can alter gene expression in later generations [94] such as the human microbiome [95]. In addition, ecological and cultural forms of inheritance are now understood to affect the behaviours and phenotypic variation of subsequent generations [96] and require inclusion into the evolutionary inheritance repertoire. Initial approaches to unify genetic and non-genetic heritability, as well as their relative contributions and mutual interactions, have successfully established quantitative models of inclusive inheritance [9]. Although non-genetic inheritance is sometimes dismissed as representing exclusively proximate mechanisms whose ultimate (evolutionary) functions do not run counter to the MS [97], the shortcomings of such arguments and of the widespread proximate–ultimate distinction in general have been convincingly demonstrated [98].

### 3.6. Niche construction

Advances at the interface of ecology, behaviour and culture have shown that populations of organisms are not merely passively exposed to natural selection but are actively involved in the formation of those environments that constitute the selective conditions for later populations. This mode of evolution, in which organisms co-direct their own evolution and that of other species, has been characterized by niche construction theory, which includes concepts of migration, dispersal and habitat selection, but also of gene–culture co-evolution. Niche construction processes can lead to the fixation of alleles that may otherwise be deleterious, it can facilitate the endurance of organisms in adverse environments and it can be beneficial despite being costly due to advantages that accrue for later generations [99].

Niche construction captures important links between biological and cultural evolution, such as the modification of selection on a plethora of human genes in response to culturally transmitted activities, the effects of which can be shown in mathematical models [20,96]. Equally encompassing effects of niche construction have been demonstrated in plants [21,69,100]. Independently of the proximate mechanism of niche construction, cultural processes can lead to the evolution and maintenance of altruistic behaviours, the emergence of high levels of cooperation, a reduction of genetic diversity or to speciation [96]. In hominin evolution, evidence has accumulated to the effect that cultural activities, such as tool-making [101] or the domestication of plants and animals [102], can be major influences on biological evolution*.* Clearly, the interconnections between biological and cultural evolution cannot be sidelined [103], and niche construction's most important theoretical contribution lies in highlighting the complex evolutionary reciprocities between organismal activity and environmental change.

### 3.7. Systems biology

From a different domain, systems biology, arise theoretical conceptions that have the capacity to integrate several of the previously mentioned evolutionary components. The kind of systems biology capable of doing this is not the ubiquitous ‘-omics’ blossoming today, but the theoretical framework that deals with the study of systems properties of organisms and their interactions across levels of organization, from molecules to populations of organisms, including physiological, behavioural and cultural factors. Although today's organismal systems biology is mostly rooted in biophysics and biological function [25], with pioneers including, among others, Ludwig von Bertalanffy, Paul Weiss, Alan Turing, D'Arcy Thompson and Claude Bernard, its endeavours are profoundly integrative, aiming at multiscale and multilevel explanations of organismal properties and their evolution. Rather than merely evoking the powers of computation for analysing multiple interactions of biological components, the capacity of systems biology is better interpreted as a scientific attitude that combines ‘reductionist’ approaches (study of constituent parts) with ‘integrationist’ approaches (study of internal and external interactions) [104]. Having gone historically through ups and downs [105], systems theoretical conceptions, whether explicitly or implicitly, now form part of the theoretical foundations of many different fields and are beginning to take centre stage in evolutionary biology also. Rupert Riedl was an early proponent of this position [106].

These examples of conceptual change in various domains of evolutionary biology represent only a condensed segment of the advances made since the inception of the MS theory some 80 years ago. Relatively minor attention has been paid to the fact that many of these concepts, which are in full use today, sometimes contradict or expand central tenets of the MS theory. Given proper attention, these conceptual expansions force us to consider what they mean for our present understanding of evolution. Obviously, several of the cornerstones of the traditional evolutionary framework need to be revised and new components incorporated into a common theoretical structure. Below, I will sketch out an expanded framework to which several of the authors in this issue have contributed.

## 4. An extended evolutionary synthesis

**Figure 2.**
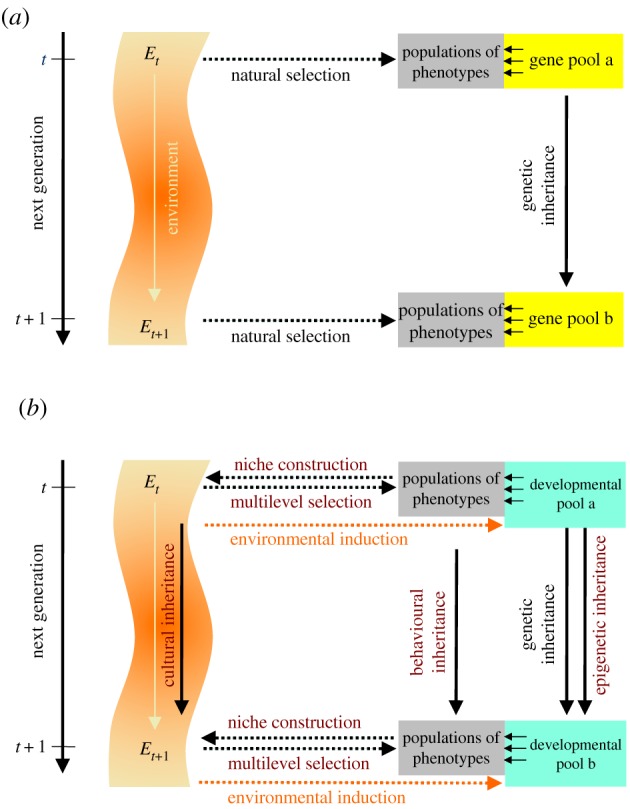
Schematic depiction of defining theory components and relations in (*a*) the MS (after Odling-Smee *et al*. [97]) and (*b*) the extended synthesis (after Müller [107]). Major differences are indicated by different colours. (Online version in colour.)

The EES was proposed as a theoretical framework that takes account of the plurality of factors and causal relations in evolutionary processes [15,49]. It continues to see variation, differential reproduction, heredity, natural selection, drift, etc., as necessary components of evolution, but it differs in how these factors are conceptualized. In addition, in the EES, development assumes a constructive role, natural selection is not the only way that variation in populations can be modified, causation does not run solely in one direction from the external environment to populations and, instead of a single inheritance mechanism, several modes of transmission exist between generations. A rough arrangement of the EES's components is depicted in figure 2 in order to visualize the structural differences between the traditional framework (figure 2*a*) and the extended framework (figure 2*b*). Several significant distinctions are noticeable. Foremost among them is the abandonment of the notion that the range of phenotypic variation in a population is sufficiently explained by the statistical correlation with concomitant variation in a population's ‘gene pool’. Instead, as the results from evo-devo and systems biology suggest, the capacity for variation in populations is determined by the developmental systems properties of a population that, in addition to genetic variation, include a host of dynamically interacting components, many of which are not genetically specified as discussed above. This may be called the ‘developmental systems pool’ of a population (figure 2*b*). Its dynamics plays out before the background of developmental plasticity and evolving gene regulation, but it also includes the self-organizing, physics-dependent and environmentally mediated properties of development. In this perspective, developmental bias and plasticity assume central roles as generators of novel and coordinated phenotypic variation by conferring directionality on the selective processes.

Inheritance is another component of the standard framework that is strongly modified in the extended picture: multiple systems of inheritance are recognized. In addition to the transmission of DNA sequences from one generation to the next, the EES includes epigenetic inheritance, in a sense that is not limited to epigenetic markings but also includes small RNAs and other maternal or paternal components as well as components of the cell that are inherited independently of the DNA. In addition, the EES accepts behavioural, ecological and cultural transmission as well as the interactions between the different modes of transgenerational inheritance. Even though the precise evolutionary contribution of each of these modes requires further study, their existence is indisputable, and distinguishing their various contributions to inclusive inheritance is essential for understanding evolutionary dynamics [9].

Natural selection remains a key factor of the EES, but its roles are reinterpreted. In the MS, at least in its bare bones interpretations, organismal shape and structure were regarded entirely as products of external selection, and the directionality of evolutionary change was supposed to result from natural selection alone. In the EES, besides the expanded range of selection to multiple levels of organization, the generative properties of developmental systems are viewed as responsible for producing phenotypic specificity, whereas natural selection serves to release that developmental potential. Particular forms of phenotypic change are taken as the result of internal generative conditions rather than external pruning. Thus, a significant amount of explanatory weight is shifted from external conditions to the internal properties of evolving populations. In addition, natural selection may be ‘bypassed’ by environmental induction, causing potentially adaptive developmental variation in many individuals of a population at once and long before natural selection may become effective.

As a consequence, unlike the MS, the EES includes a constructive component. Instead of chance variation in DNA composition, evolving developmental interactions account for the specificities of phenotypic construction. This interpretation is also based on a fundamentally different account of the role of genes in development and evolution. In the EES, genes are not causally privileged as programs or blueprints that control and dictate phenotypic outcomes, but are rather parts of the systemic dynamics of interactions that mobilize self-organizing processes in the evolution of development and entire life cycles. This represents a shift from a *programmed* to a *constructive* role of developmental processes in evolution. Furthermore, the constructive aspect also concerns the interactions between all other levels of organization such as the behavioural, social and cultural. Together, they constitute the kernel of an organizational theory component that sets the EES apart from the MS.

Another distinctive feature of the EES is causal reciprocity. This is true for two domains. One is the construction of phenotypic complexity, in which causation not only flows from the lower levels of biological organization, such as DNA, ‘upwards’ to cells, tissues and organisms, but also from the higher level ‘downwards’, such as through environmental- or tissue-induced gene regulation. The second aspect of causal reciprocity lies in the fact that populations of organisms are not relegated to being passive recipients of external selection pressures but, through various forms of niche construction, actively modify the environments that become the selective conditions for later generations. Thus, a major feature of the EES is that causation not only runs one way but assumes dialectical relations between its participating components, both in the relationship of populations with the environment and in the generation of heritable phenotypic architectures.

The novelty of the EES and the differences with the MS theory become most apparent in the predictions that derive from the EES framework, both with regard to short-term and long-term effects of organismal evolution. The most important predictions concern the following: (i) the generation of heritable phenotypic variation (variation will be systematically biased and facilitated by the generative features of development); (ii) the origin of phenotypic novelty (novelties are due to emergent and self-organizing properties of developmental systems); (iii) the sequence of genetic and phenotypic change (emergent phenotypic structures can be captured and stabilized by evolving gene regulatory circuitry and assume fitness subsequently); (iv) inheritance (in addition to genetic inheritance, adaptive variants are propagated by non-genetic inheritance, learning and cultural transmission, as well as by repeated environmental induction); (v) tempo of evolution (periods of rapid phenotypic evolution can alternate with periods of slow and continuous change); (vi) environmental induction (phenotypic variation can be environmentally induced in multiple individuals simultaneously); (vii) organismal activity (niche construction effectuates environmental changes that enhance the fitness of the constructors and their descendants; (viii) natural selection (the primary evolutionary effect of natural selection is not to eliminate the unfit but to release generative potential).

Overall, the EES proposes that variation is more predictable and selection effects are less directional than hitherto argued. The EES addresses organizing principles instead of statistical correlations or evolving instruction programs. It represents a pluralistic, process-based framework of dynamical interactions between a multitude of evolutionarily effective factors and generates its own set of evolutionary predictions that make it clearly distinct from the MS account. These genuine predictions of the EES give rise to new research programmes, which have already generated validating empirical results. It is beyond the scope of this article to discuss the range of predictions and their consequences in greater detail, but more extensive treatments can be found in Laland *et al*. [15].

## 5. Consequences

The EES is not a simple, unfounded call for a new theory but has become an ongoing project for integrating the theoretically relevant concepts that have arisen from multiple fields of evolutionary biology. Although the EES recognizes the fundaments of the classical MS theory, it differs in its interpretation of the role of some of its elements and integrates new components, such as constructive processes of development, multiple inheritance mechanisms, niche reciprocity, as well as behavioural and cultural elements (on which this overview did not dwell much, but see other contributions to this issue). It is unavoidable to notice that an integration of these concepts means not a simple add-on of a few peripheral notions to the MS model without any effects on its core logic. Rather, the EES establishes a new structure of the theoretical evolutionary framework that goes beyond the reductionist and gene-centred perspective of the past. It represents a different way of thinking about evolution, historically rooted in the organicist tradition [108]. Its predictions permit the derivation of new hypotheses and thus inspire novel and progressive research in evolutionary biology and adjacent fields.

Proposals of an EES generally elicit rather positive reactions from the representatives of different fields of science, many of whom are convinced that an expanded theoretical framework has become necessary for evolutionary biology. Opposition comes in three different versions. One is the ‘absorption argument’, i.e. the standard framework is said to no longer be the MS but to have continually absorbed various conceptual advances [33]. The defenders of the EES beg to differ: as long as the major predictions that can be derived from an evolutionary framework remain exactly those of the classical MS, no change to its core assumptions has happened. Adding a chapter or two on new domains of evolutionary research, as evolution textbooks increasingly do, does not mean that these concepts have been integrated into the theoretical edifice of evolutionary biology. Rather, it has become customary to treat individual research questions independently, but to accept only the population genetic approach as explanatorily essential.

The second response (also made by a participant after nearly every lecture in the Royal Society meeting on which this special issue is based) runs: ‘this has been said before’, implying either that the arguments are outdated or deemed irrelevant. It remains unclear why empirical findings or conceptual proposals that have been stated previously are thereby rendered irrelevant. When this objection fails, the argument usually becomes that the processes central to the EES are merely add-ons to the basic processes required by the MS, such as natural selection, mutation, recombination, drift and gene flow, but are ‘not essential’ for evolution [33]. Given the different explananda of evolutionary biology described above, such suggestions are beside the point. Moreover, critics infallibly call for further empirical evidence, giving the impression that the EES is an unfounded theoretical exercise that still awaits confirmation. While more empirical evidence is always desirable, all constituent parts of the EES are already abundantly supported by research results in the different domains from which they have arisen, as amply shown by the works cited in the present overview. Ideas perhaps rightly rejected in the past due to a lack of supporting evidence must now be re-evaluated in the light of contemporary knowledge.

A subtler version of the this-has-been-said-before argument used to deflect any challenges to the received view is to pull the issue into the never ending micro-versus-macroevolution debate. Whereas ‘microevolution’ is regarded as the continuous change of allele frequencies within a species or population [109], the ill-defined macroevolution concept [36], amalgamates the issue of speciation and the origin of ‘higher taxa’ with so-called ‘major phenotypic change’ or new constructional types. Usually, a cursory acknowledgement of the problem of the origin of phenotypic characters quickly becomes a discussion of population genetic arguments about speciation, often linked to the maligned punctuated equilibria concept [9], in order to finally dismiss any necessity for theory change. The problem of phenotypic complexity thus becomes (in)elegantly bypassed. Inevitably, the conclusion is reached that microevolutionary mechanisms are consistent with macroevolutionary phenomena [36], even though this has very little to do with the structure and predictions of the EES. The real issue is that genetic evolution alone has been found insufficient for an adequate causal explanation of all forms of phenotypic complexity, not only of something vaguely termed ‘macroevolution’. Hence, the micro–macro distinction only serves to obscure the important issues that emerge from the current challenges to the standard theory. It should not be used in discussion of the EES, which rarely makes any allusions to macroevolution, although it is sometimes forced to do so.

Interestingly, a third class of responses to the EES is this: the proposed modifications are not radical enough, a much more fundamental change is required [107]. Also, here we beg to differ. Quite evidently, the MS theory has become too narrow in several regards, but this does not mean that all its elements have been invalidated. Nevertheless, the differences in structure and consequences are substantial enough to require a new designation, because to continue using ‘MS’ evokes a wholly different set of assumptions and predictions. The classical theory cannot keep its label and at the same time make different predictions. The term ‘EES’ used here and elsewhere [4,5,9,14,15,27,28,49] is not meant as a simple extension *of* the MS, as sometimes wrongly implied, but to indicate a comprehensive new synthesis. Whether eventually that new framework will be called EES or a different name is not important. What is important is that a different theory structure is necessary to accommodate the new concepts that are in everyday use and have become part of the current toolkit of evolutionary biology. Therefore, a theory change is not a future goal, but we are in the midst of it, with the EES attempting to provide a structure for the present state of evolutionary thought.

This is an exciting period in evolutionary biology. The principal Darwinian research tradition is upheld, but the specifics of evolutionary theory structure are undergoing ferment, including the revision of some of its traditional elements and the incorporation of new elements. Instead of privileging selected mechanisms such as random variation, genetic control and natural selection, the multitude of factors that dynamically interact in the evolutionary process will be better expounded by a pluralistic theory framework. Current evolutionary research already reflects this pluralism, and as many of its underlying concepts have drifted from the standard theoretical paradigm, an adjusted evolutionary framework that adequately synthesizes the multitude of new theoretical elements has become a necessity. The EES represents one possibility for such integration.

## Data accessibility

This article has no additional data.
